# Crystal structure of *catena*-poly[[di­aqua­di­imida­zole­cobalt(II)]-μ_2_-2,3,5,6-tetra­bromo­benzene-1,4-di­carboxyl­ato]

**DOI:** 10.1107/S2056989024009915

**Published:** 2024-10-31

**Authors:** Hitoshi Kumagai, Satoshi Kawata, Nobuhiro Ogihara

**Affiliations:** a Toyota Central R&D Labs., Inc., 41-1 Yokomichi, Nagakute, Aichi 480-1192, Japan; b Department of Chemistry Fukuoka University 8-19-1 Nanakuma Jonan-ku Fukuoka 814-0180 Japan; Tokyo University of Science, Japan

**Keywords:** crystal structure, cobalt, hydrogen bonding, tetra­bromo­terephthalate, imidazole

## Abstract

The asymmetric unit of the title compound, [Co(C_8_Br_4_O_4_)(C_3_H_4_N_2_)_2_(H_2_O)_2_]_*n*_ or [Co(Br_4_bdc)(im)_2_(H_2_O)_2_]_*n*_, comprises half of Co^II^ ion, tetra­bromo­benzene­dicarboxylate (Br_4_bdc^2−^), imidazole (im) and a water mol­ecule. The Co^II^ ion exhibits a six-coordinated octa­hedral geometry with two oxygen atoms of the Br_4_bdc^2−^ ligand, two oxygen atoms of the water mol­ecules, and two nitro­gen atoms of the im ligands. The carboxyl­ate group is nearly perpendicular to the benzene ring and shows monodentate coordination to the Co^II^ ion. The Co^II^ ions are bridged by the Br_4_bdc^2−^ ligand, forming a one-dimensional chain. The carboxyl­ate group acts as an inter­molecular hydrogen-bond acceptor toward the im ligand and a coordinated water mol­ecule. The chains are connected by inter­chain N—H⋯O(carboxyl­ate) and O—H(water)⋯O(carboxyl­ate) hydrogen-bonding inter­actions and are not arranged in parallel but cross each other *via* inter­chain hydrogen bonding and π–π inter­actions, yielding a three-dimensional network.

## Abstract 2

In [Co(Br_4_bdc)(im)_2_(H_2_O)_2_], the Co^II^ ions are bridged by the Br_4_bdc^2−^ ligand, forming one-dimensional chains, which are inter­connected by inter­chain N–H⋯O and O–H⋯O hydrogen-bonding and π–π inter­actions, yielding a three-dimensional network.

## 1. Chemical context

Infinite assemblies of metal ions bridged by organic linkers, so-called metal–organic frameworks (MOFs) or coordination polymers (CPs), are being actively investigated (Cheetham *et al.*, 1999 ▸; Férey, 2008 ▸; Kitagawa *et al.*, 2004 ▸; Rao *et al.*, 2008 ▸; Yaghi, *et al.*, 2019 ▸). Benzene­dicarboxyl­ate (bdc^2−^ dianion), also known as terephthalate dianion, is a well-known linker that gives functional MOFs or CPs (Eddaoudi *et al.*, 2002 ▸; Kurmoo 2009 ▸). We have not only been preparing electrode materials using terephthalate dianion and its analogues (Ogihara *et al.*, 2014 ▸, 2017 ▸, 2023 ▸; Yasuda *et al.*, 2014 ▸; Mikita *et al.*, 2020 ▸) but also fine tuning the crystal structures and properties of MOFs and CPs using *R*_4_bdc^2−^ dianions (*R* = H, F, Cl, Br) in which halogen atoms and metal ions are systematically varied (Kumagai *et al.*, 2012 ▸, 2021 ▸). We have used 4,4′-bi­pyridine (4,4′-bpy) or pyrazine (pyz) as co-ligands and have reported on the structure, thermal stability, and water adsorption/desorption properties of the resultant materials. In this contribution, we focused on using the Br_4_bdc^2−^ dianion and imidazole (im) as a co-ligand instead of a pyz ligand in the synthesis of a Co^II^–Br_4_bdc^2−^ dianion system to observe the structural change resulting from the substitution of im for pyz. Although the pyz ligand coordinates two metal centers linearly, one of the two nitro­gen atoms of the im ligand is protonated and undergoes hydrogen-bonding inter­actions. Here, we report on the single-crystal structure and properties of [Co(Br_4_bdc)(im)_2_(H_2_O)_2_]. This is the first structural characterization of a metal complex having the Br_4_bdc^2−^ dianion and im as a co-ligand.
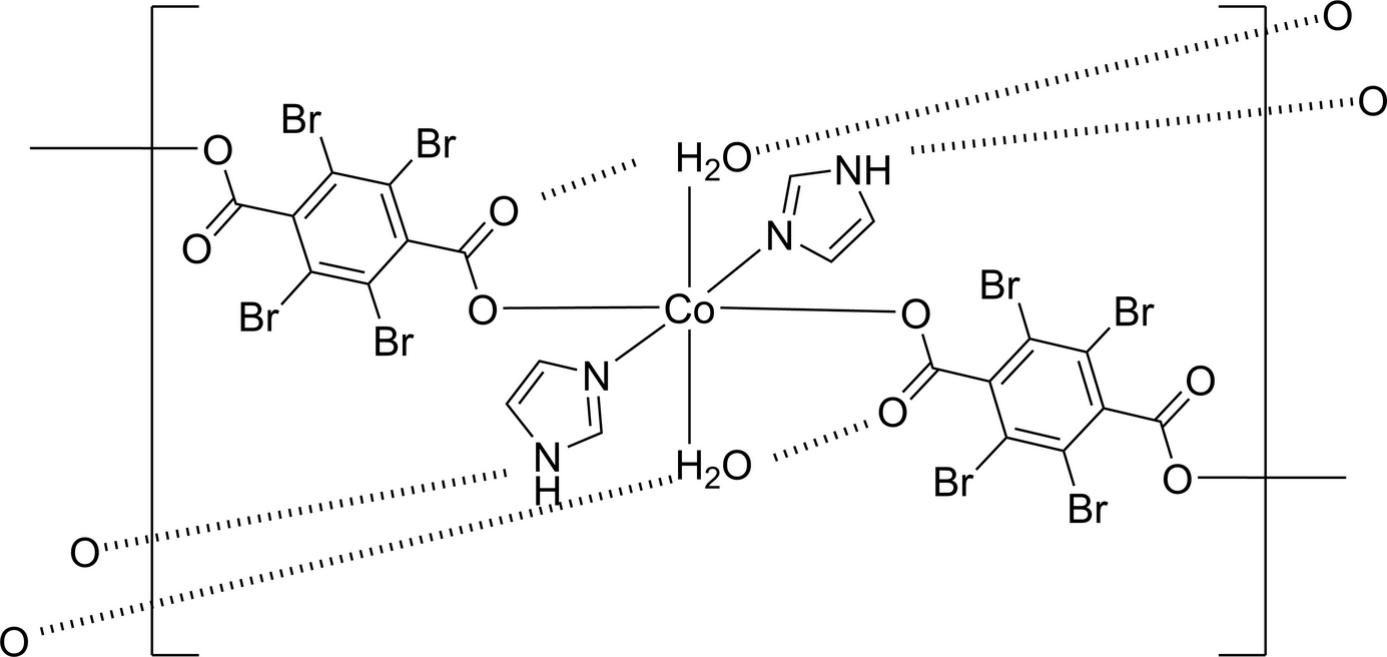


## 2. Structural commentary

**Figure 1:**
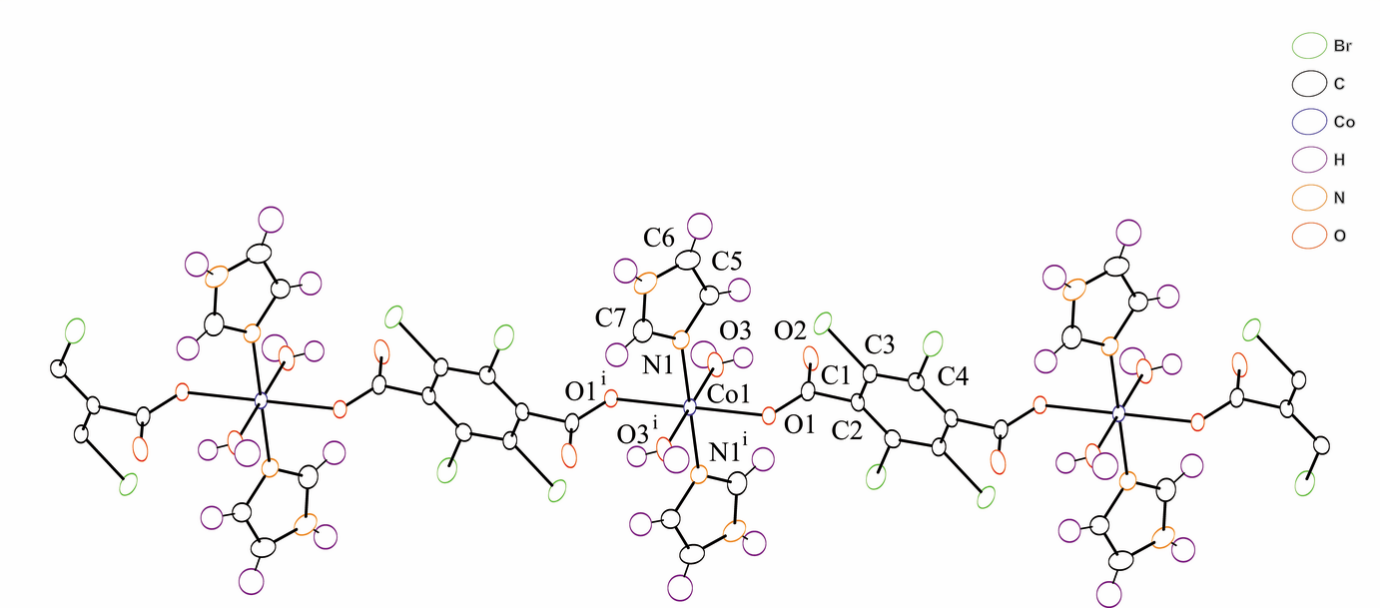
One-dimensional chain structure of title compound, along with labeling scheme and 50% probability displacement ellipsoids. [Symmetry code: (i) −*x* + 1, −*y* + 2, −*z* + 2.]

The title compound, [Co(Br_4_bdc)(im)_2_(H_2_O)_2_], consists of a Co^II^ ion, a tetra­bromo­benzene­dicarboxyl­ate dianion (Br_4_bdc^2−^), two imidazole (im) mol­ecules, and two water mol­ecules. Its asymmetric unit consists of half of a Co^II^ ion, half of a Br_4_bdc^2−^ dianion, an im mol­ecule, and a water mol­ecule. The key feature of the structure is a three-dimensional (3D) hydrogen-bonding network that consists of one-dimensional (1D) coordination chains built up by CoO_4_N_2_ octa­hedra bridged by Br_4_bdc^2−^ ligands and inter­chain N—H⋯O and O—H⋯O hydrogen-bonding inter­actions. Fig. 1 ▸ shows the chain structure of [Co(Br_4_bdc)(im)_2_(H_2_O)_2_]. The Co^II^ ion occupies a crystallographically special position, and each pair of Br_4_bdc^2−^ ligands, water mol­ecules, and im ligands coordinates *trans* to each other; the coordination environment is similar to that of a two-dimensional (2D) material synthesized from Br_4_bdc^2−^ and pyz ligands, [Co(Br_4_bdc)(pyz)_2_(H_2_O)_2_] (Kumagai *et al.*, 2021 ▸). The carboxyl­ate group exhibits a monodentate coordination, and the benzene ring and the carboxyl­ate group are nearly perpendicular dihedral angle = 90.5 (3)°]. The im ligand coordinates to the Co^II^ ion *via* nitro­gen atom N1 as a neutral imidazole ligand rather than as an imidazolate anion, and a hydrogen atom is attached to the remaining nitro­gen atom N2. The Co—O3 (H_2_O) and Co—O1 (carboxyl­ate) bond lengths in the title compound are 2.1006 (16) and 2.1678 (13) Å (Table 1 ▸), respectively, which are slightly longer than those [2.032 (3) Å and 2.096 (4) Å] for [Co(Br_4_bdc)(pyz)_2_(H_2_O)_2_] (Kumagai *et al.*, 2021 ▸). However, the Co—N bond length of 2.0850 (18) Å is shorter than that of 2.273 (4) Å for [Co(Br_4_bdc)(pyz)_2_(H_2_O)_2_], in which the Co^II^ ion shows an elongated octa­hedral environment, indicative of the compressed octa­hedron of the title compound (Kumagai *et al.*, 2021 ▸). The angles around the Co^II^ ion lie in the range 88.31 (6)–180.0°. The Co⋯Co separation defined by the Co–Br_4_bdc^2−^–Co connectivity within the chain is 11.69 Å, which is slightly longer than that for [Co(Br_4_bdc)(pyz)_2_(H_2_O)_2_] (11.24 Å; Kumagai *et al.*, 2021 ▸).

## 3. Supra­molecular features

**Figure 2:**
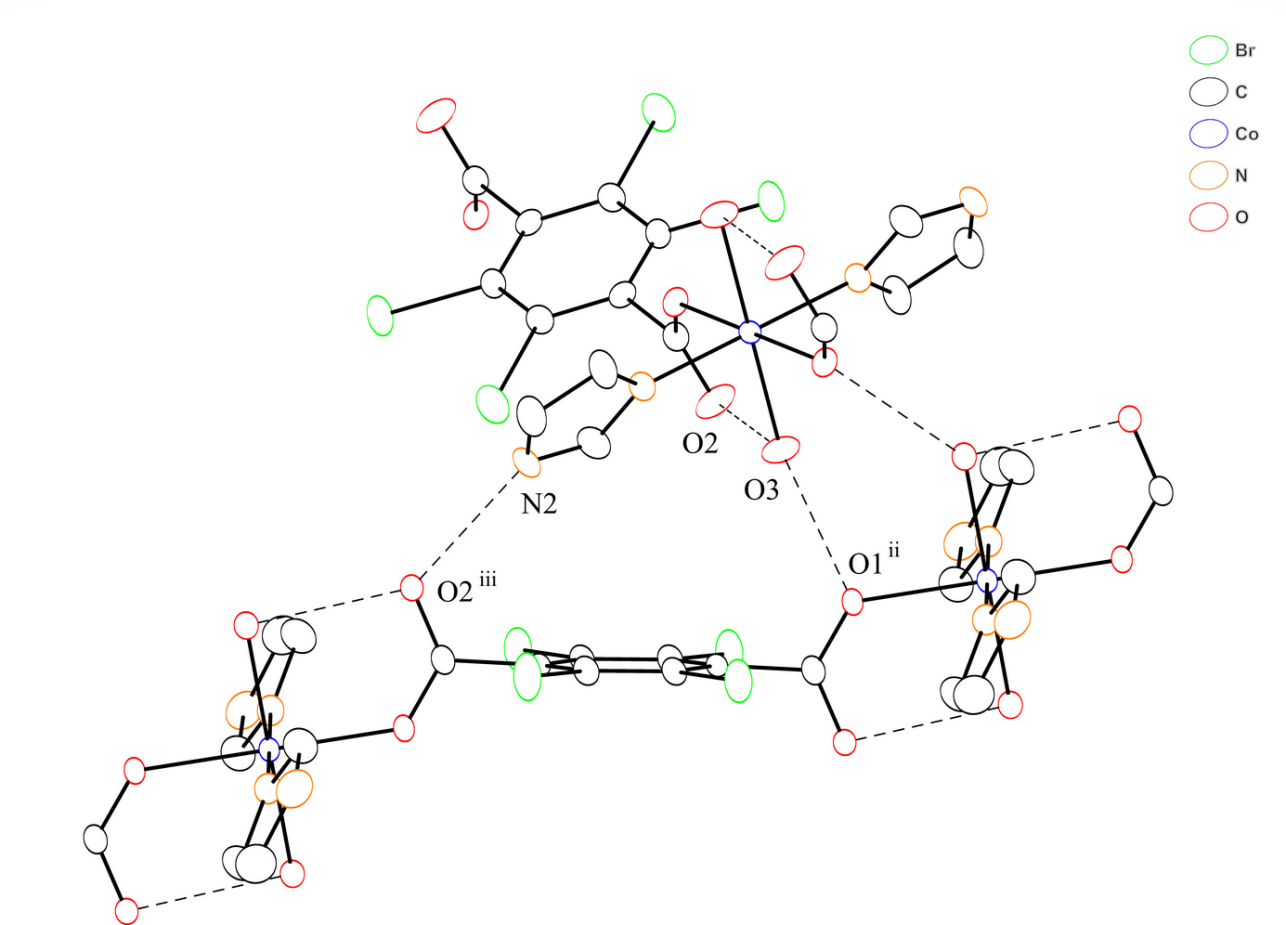
View of inter- and intra­molecular hydrogen-bonding inter­actions. Dashed lines represent hydrogen bonds. Hydrogen atoms are omitted for clarity. [Symmetry code: (ii) *x*, −*y* + 2, *z* + 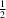, (iii) −*x* + 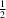, *y* − 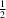, −*z* + 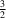.]

**Figure 3:**
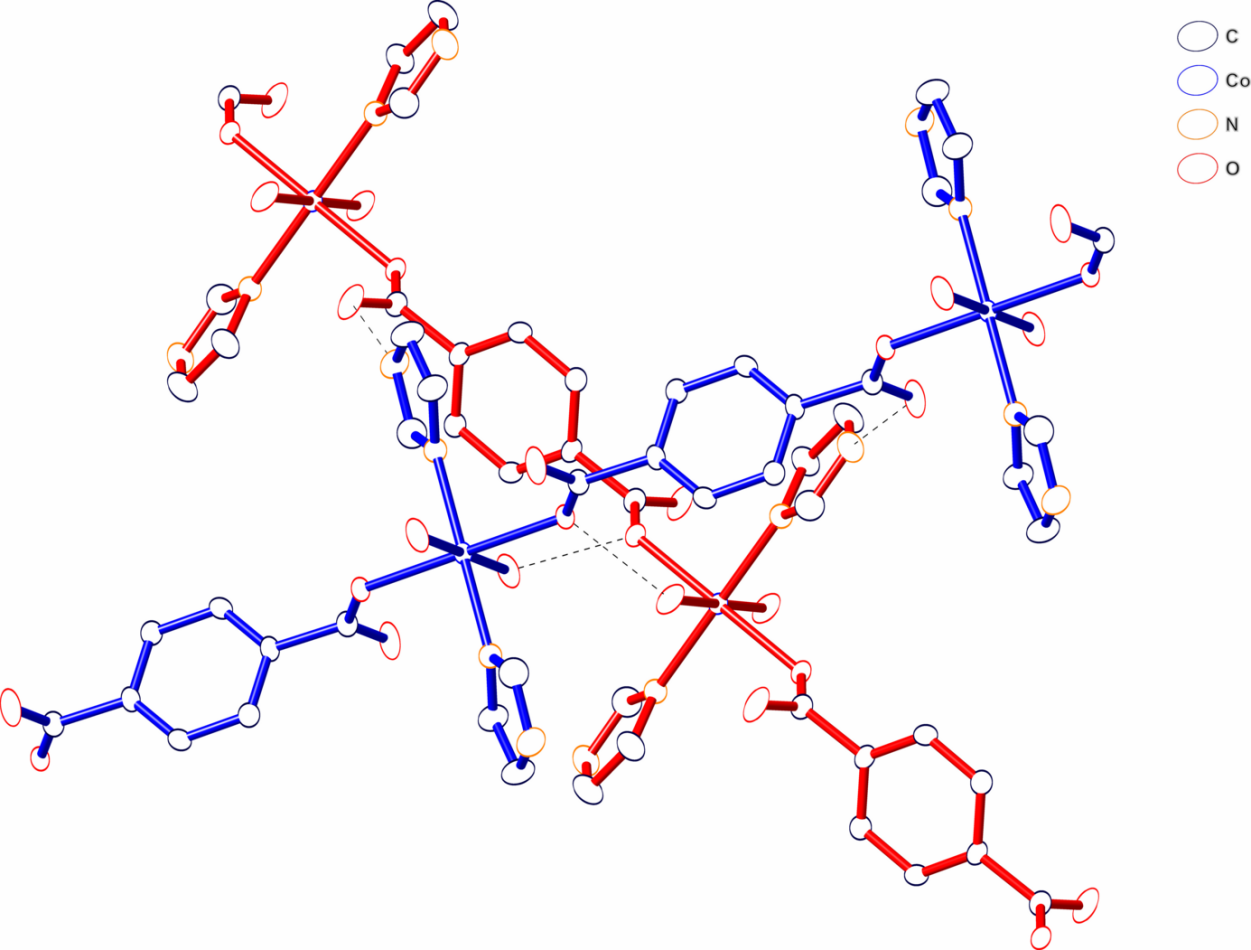
Two chains linked by inter­chain hydrogen-bonding inter­actions. Dashed lines represent inter­chain hydrogen bonds. Bromine and hydrogen atoms are omitted for clarity.

**Figure 4:**
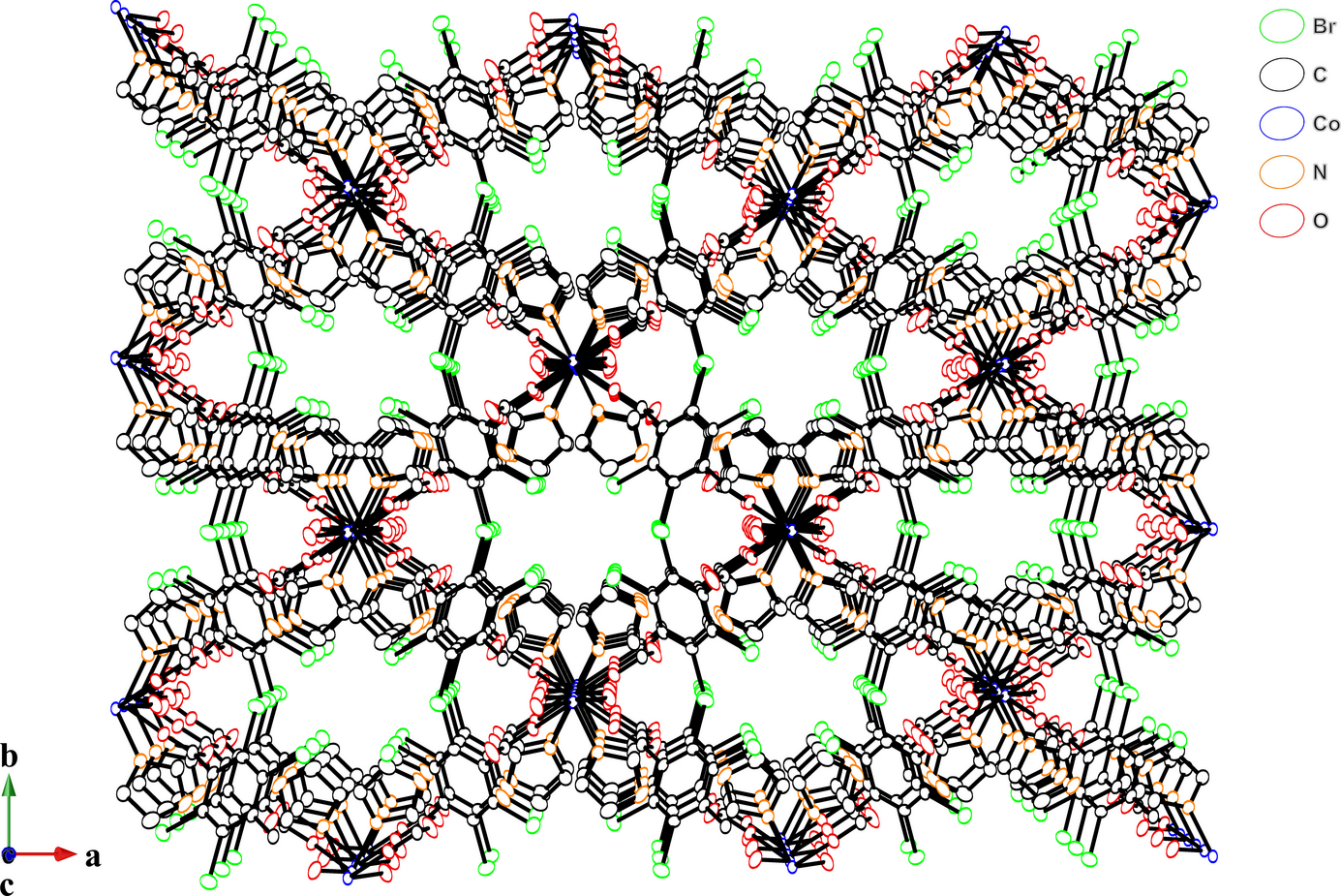
View of the hydrogen-bonding network along the crystallographic *c* axis.

In the crystal structure, the im ligands and coordinated water mol­ecules act as hydrogen-bond donors and the oxygen atoms of the carboxyl­ate group of Br_4_bdc^2−^ ligand act as hydrogen-bond acceptors (Fig. 2 ▸, Table 2 ▸). The coordinated water mol­ecule exhibits not only intra­chain hydrogen-bonding inter­actions with oxygen atom O2 of the carboxyl­ate group not bound to a Co^II^ ion but also inter­chain hydrogen-bonding inter­actions with the coordinated oxygen atom O1 of carboxyl­ate group in the adjacent chain. Further inter­chain hydrogen-bonding inter­actions between the im ligands and coordinated oxygen atoms of the carboxyl­ate groups yield a 3D hydrogen-bonded network. The nearest centroid–centroid distance between the benzene ring and the im ligand, and the shortest C⋯C distance are 3.95 and 3.63 Å, respectively. These distances are indicative of some degree of π–π stacking inter­actions (Kruszynski & Sierański, 2019 ▸). The chains are not arranged in parallel but cross each other by hydrogen-bonding and π–π stacking inter­actions (Fig. 3 ▸) and appear to have a 1D-channel structure when viewed along the *c* axis (Fig. 4 ▸).

## 4. Database survey

Although a search of the Sci Finder database for structures with a Br_4_bdc^2−^ ion, an im ligand, and a Co^II^ ion resulted in no complete matches, nor were any partially matched structures found. They are metal complexes composed of a Br_4_bdc^2−^ ligand and benzimidazole derivatives (Zhang *et al.*, 2016 ▸; Hu *et al.*, 2015 ▸). A search of the Web of Science database for the keyword tetra­bromo­terephthalate led to Ni^II^ compounds that also contain a Br_4_bdc^2−^ ligand and benzimidazole derivatives (Liu *et al.*, 2015 ▸; Hao *et al.*, 2020 ▸).

## 5. Synthesis and crystallization

An aqueous solution (5 mL) of cobalt(II) nitrate hexa­hydrate (0.35 g, 1.25 mmol) was transferred to a glass tube, and an ethanol–water mixture (5 mL) of 2,3,5,6-tetra­bromo­benzene­dicarb­oxy­lic acid (0.60 g, 1.25 mmol), NaOH (0.10 g, 2.50 mmol), and imidazole (0.10 g, 1.25 mmol) was poured into the glass tube without the two solutions being mixed. Pink crystals began to form at ambient temperature in 1 week, one of which was used for the X-ray crystallography study.

## 6. Refinement

Crystal data, data collection, and structure refinement details are summarized in Table 3 ▸. The non-hydrogen atoms were refined anisotropically. The hydrogen atom attached to a nitro­gen atom of the im ligand and the water mol­ecules were located in difference-Fourier maps. Other hydrogen atoms were placed in idealized positions and were refined using a riding model.

## 7. Additional investigations

To assess the thermal properties of the title compound, we carried out a thermogravimetric analysis (TGA) under a nitro­gen atmosphere. The TGA curve is characterized by two weight-loss steps in the range 120–500°C (Fig. S1). The first weight loss of 6% was observed in the temperature range 120–160°C, and the second weight loss of 90% was observed in the range 200–470°C. The first weight loss corresponds to the loss of two coordinated water mol­ecules to give the dehydrated phase, [Co(Br_4_bdc)(im)_2_]. The second weight loss is due to thermal decomposition of the compound. The DTG curve exhibited a sharp peak at 215°C. This result indicates that the compound is stable up to about 200°C. Electronic diffuse-reflectance spectra were recorded for as-synthesized [Co(Br_4_bdc)(im)_2_(H_2_O)_2_] and the dehydrated phase, [Co(Br_4_bdc)(im)_2_], obtained after heat treatment at 140°C. Because the compounds were not soluble in any solvent, we acquired the electronic diffuse-reflectance spectra of solid-state samples. After the heat treatment, a color change from pink to blue was observed and two strong absorption bands appeared at ∼480 nm and ∼1000 nm, indicating that the coordination environment of the six-coordinate Co^II^ center had changed to a four-coordinate Co^II^ environment as a result of the loss of coordinated water mol­ecules (Fig. S2). In the IR spectrum, the characteristic band for the coordinated water mol­ecules was observed as a broad band at 3340 cm^−1^; the band disappeared after the heat treatment (Fig. S3). This result is in good agreement with the TGA and electronic diffuse-reflectance spectra measurement results. Nitro­gen adsorption–desorption measurements were conducted; however, almost no nitro­gen was adsorbed. This lack of nitro­gen adsorption is speculatively attributed to insufficient space for nitro­gen mol­ecules because of the large ionic radius of the bromine.

## Supplementary Material

Crystal structure: contains datablock(s) I. DOI: 10.1107/S2056989024009915/jp2013sup1.cif

Structure factors: contains datablock(s) I. DOI: 10.1107/S2056989024009915/jp2013Isup2.hkl

Supporting information file. DOI: 10.1107/S2056989024009915/jp2013Isup3.cdx

Supplementary figures. DOI: 10.1107/S2056989024009915/jp2013sup4.pdf

CCDC reference: 2391124

Additional supporting information:  crystallographic information; 3D view; checkCIF report

## Figures and Tables

**Table 1:** Selected geometric parameters (Å, °)

Co1—N1^i^	2.0850 (18)	Co1—O3	2.1007 (16)
Co1—N1	2.0850 (18)	Co1—O1^i^	2.1678 (13)
Co1—O3^i^	2.1006 (16)	Co1—O1	2.1678 (13)
			
N1^i^—Co1—N1	180.00 (11)	O3^i^—Co1—O1^i^	89.81 (6)
N1^i^—Co1—O3^i^	89.06 (7)	O3—Co1—O1^i^	90.19 (6)
N1—Co1—O3^i^	90.94 (7)	N1^i^—Co1—O1	91.69 (6)
N1^i^—Co1—O3	90.94 (7)	N1—Co1—O1	88.31 (6)
N1—Co1—O3	89.06 (7)	O3^i^—Co1—O1	90.19 (6)
O3^i^—Co1—O3	180.00 (4)	O3—Co1—O1	89.81 (6)
N1^i^—Co1—O1^i^	88.31 (6)	O1^i^—Co1—O1	180.0
N1—Co1—O1^i^	91.69 (6)		

Symmetry code: (i) 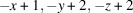.

**Table 2:** Hydrogen-bond geometry (Å, °)

*D*—H⋯*A*	*D*—H	H⋯*A*	*D*⋯*A*	*D*—H⋯*A*
N2—H1⋯O2^ii^	0.80 (3)	2.01 (3)	2.808 (2)	174 (3)
O3—H2⋯O2	0.76 (3)	1.99 (3)	2.700 (2)	155 (3)
O3—H3⋯O1^iii^	0.75 (4)	2.06 (4)	2.809 (2)	176 (4)

Symmetry codes: (ii) 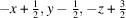; (iii) 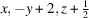.

**Table 3:** Experimental details

Crystal data	
Chemical formula	[Co(C_8_Br_4_O_4_)(C_3_H_4_N_2_)_2_(H_2_O)_2_]
*M*_r_	710.85
Crystal system, space group	Monoclinic, *C*2/*c*
Temperature (K)	173
*a*, *b*, *c* (Å)	18.8050 (7), 12.2925 (6), 10.8938 (5)
β (°)	121.853 (3)
*V* (Å^3^)	2138.97 (17)
*Z*	4
Radiation type	Mo *K*α
μ (mm^−1^)	8.31
Crystal size (mm)	0.60 × 0.60 × 0.60

Data collection	
Diffractometer	Rigaku R-AXIS RAPID
Absorption correction	Multi-scan (*ABSCOR*; Rigaku, 1995 ▸)
*T*_min_, *T*_max_	0.004, 0.007
No. of measured, independent and observed [*I* > 2σ(*I*)] reflections	10419, 2448, 2277
*R*_int_	0.046
(sin θ/λ)_max_ (Å^−1^)	0.649

Refinement	
*R*[*F*^2^ > 2σ(*F*^2^)], *wR*(*F*^2^), *S*	0.028, 0.067, 1.07
No. of reflections	2448
No. of parameters	145
H-atom treatment	H atoms treated by a mixture of independent and constrained refinement
Δρ_max_, Δρ_min_ (e Å^−3^)	0.61, −0.60

Computer programs: RAPID AUTO (Rigaku, 1995 ▸), *SUPERFLIP* (Palatinus & Chapuis, 2007 ▸), *SHELXL2018/3* (Sheldrick, 2015 ▸) and *CrystalStructure* (Rigaku, 2019 ▸).

## supplementary crystallographic information

### catena-Poly[[diaquadiimidazolecobalt(II)]-µ2-2,3,5,6-tetrabromobenzene-1,4-dicarboxylato] Crystal data

**Table:**

[Co(C_8_Br_4_O_4_)(C_3_H_4_N_2_)_2_(H_2_O)_2_]	*F*(000) = 1356
*M**_r_* = 710.85	*D*_x_ = 2.207 Mg m^−^^3^
Monoclinic, *C*2/*c*	Mo *K*α radiation, λ = 0.71075 Å
*a* = 18.8050 (7) Å	Cell parameters from 9142 reflections
*b* = 12.2925 (6) Å	θ = 3.3–27.5°
*c* = 10.8938 (5) Å	µ = 8.31 mm^−^^1^
β = 121.853 (3)°	*T* = 173 K
*V* = 2138.97 (17) Å^3^	Block, pink
*Z* = 4	0.60 × 0.60 × 0.60 mm

### catena-Poly[[diaquadiimidazolecobalt(II)]-µ2-2,3,5,6-tetrabromobenzene-1,4-dicarboxylato] Data collection

**Table:**

Rigaku R-AXIS RAPID diffractometer	2277 reflections with *I* > 2σ(*I*)
ω scans	*R*_int_ = 0.046
Absorption correction: multi-scan (ABSCOR; Rigaku, 1995)	θ_max_ = 27.5°, θ_min_ = 3.3°
*T*_min_ = 0.004, *T*_max_ = 0.007	*h* = −22→24
10419 measured reflections	*k* = −15→15
2448 independent reflections	*l* = −14→14

### catena-Poly[[diaquadiimidazolecobalt(II)]-µ2-2,3,5,6-tetrabromobenzene-1,4-dicarboxylato] Refinement

**Table:**

Refinement on *F*^2^	Primary atom site location: structure-invariant direct methods
Least-squares matrix: full	Secondary atom site location: difference Fourier map
*R*[*F*^2^ > 2σ(*F*^2^)] = 0.028	Hydrogen site location: mixed
*wR*(*F*^2^) = 0.067	H atoms treated by a mixture of independent and constrained refinement
*S* = 1.07	*w* = 1/[σ^2^(*F*_o_^2^) + (0.0356*P*)^2^ + 0.8085*P*] where *P* = (*F*_o_^2^ + 2*F*_c_^2^)/3
2448 reflections	(Δ/σ)_max_ = 0.002
145 parameters	Δρ_max_ = 0.61 e Å^−^^3^
0 restraints	Δρ_min_ = −0.59 e Å^−^^3^

### catena-Poly[[diaquadiimidazolecobalt(II)]-µ2-2,3,5,6-tetrabromobenzene-1,4-dicarboxylato] Special details

**Table:**

Geometry. All esds (except the esd in the dihedral angle between two l.s. planes) are estimated using the full covariance matrix. The cell esds are taken into account individually in the estimation of esds in distances, angles and torsion angles; correlations between esds in cell parameters are only used when they are defined by crystal symmetry. An approximate (isotropic) treatment of cell esds is used for estimating esds involving l.s. planes.

### catena-Poly[[diaquadiimidazolecobalt(II)]-µ2-2,3,5,6-tetrabromobenzene-1,4-dicarboxylato] Fractional atomic coordinates and isotropic or equivalent isotropic displacement parameters (Å^2^)

**Table:**

	*x*	*y*	*z*	*U*_iso_*/*U*_eq_	
Br1	0.40878 (2)	1.36557 (2)	0.78290 (2)	0.02796 (9)	
Br2	0.30071 (2)	1.49911 (2)	0.46852 (3)	0.03101 (10)	
Co1	0.500000	1.000000	1.000000	0.01141 (10)	
O1	0.40770 (8)	1.08127 (12)	0.80246 (14)	0.0157 (3)	
O2	0.31711 (10)	1.14631 (14)	0.85848 (17)	0.0282 (4)	
O3	0.41853 (11)	1.01046 (14)	1.0767 (2)	0.0236 (4)	
N1	0.44446 (11)	0.85236 (14)	0.90200 (19)	0.0179 (3)	
N2	0.34569 (13)	0.73172 (17)	0.7762 (2)	0.0275 (4)	
C1	0.34466 (12)	1.13493 (16)	0.7774 (2)	0.0159 (4)	
C2	0.29633 (12)	1.19335 (16)	0.6314 (2)	0.0153 (4)	
C3	0.31735 (12)	1.29953 (17)	0.6185 (2)	0.0159 (4)	
C4	0.27151 (13)	1.35524 (16)	0.4882 (2)	0.0167 (4)	
C5	0.47955 (14)	0.76163 (19)	0.8814 (3)	0.0254 (5)	
H5	0.537318	0.752777	0.915853	0.030*	
C6	0.41879 (16)	0.6861 (2)	0.8039 (3)	0.0329 (6)	
H6	0.425979	0.616103	0.775114	0.039*	
C7	0.36370 (14)	0.8307 (2)	0.8368 (2)	0.0257 (5)	
H7	0.323364	0.879232	0.833300	0.031*	
H1	0.2999 (19)	0.706 (2)	0.733 (3)	0.036 (8)*	
H2	0.383 (2)	1.050 (3)	1.028 (3)	0.032 (8)*	
H3	0.418 (2)	0.987 (2)	1.139 (4)	0.040 (9)*	

### catena-Poly[[diaquadiimidazolecobalt(II)]-µ2-2,3,5,6-tetrabromobenzene-1,4-dicarboxylato] Atomic displacement parameters (Å^2^)

**Table:**

	*U*^11^	*U*^22^	*U*^33^	*U*^12^	*U*^13^	*U*^23^
Br1	0.02444 (15)	0.02595 (15)	0.01768 (14)	−0.00432 (8)	0.00031 (11)	−0.00049 (8)
Br2	0.03257 (17)	0.02081 (15)	0.02504 (16)	−0.00629 (8)	0.00522 (13)	0.00651 (8)
Co1	0.00890 (19)	0.0133 (2)	0.0105 (2)	0.00010 (12)	0.00411 (16)	0.00132 (12)
O1	0.0144 (7)	0.0176 (7)	0.0121 (7)	0.0044 (5)	0.0050 (6)	0.0030 (5)
O2	0.0253 (8)	0.0442 (10)	0.0199 (8)	0.0198 (7)	0.0153 (7)	0.0143 (7)
O3	0.0199 (8)	0.0337 (10)	0.0229 (9)	0.0100 (7)	0.0151 (8)	0.0135 (7)
N1	0.0168 (8)	0.0168 (9)	0.0171 (8)	−0.0012 (7)	0.0069 (7)	0.0008 (6)
N2	0.0229 (10)	0.0309 (11)	0.0267 (11)	−0.0149 (8)	0.0116 (9)	−0.0068 (8)
C1	0.0126 (9)	0.0171 (10)	0.0137 (10)	0.0012 (7)	0.0039 (8)	0.0018 (7)
C2	0.0128 (9)	0.0188 (10)	0.0144 (9)	0.0051 (8)	0.0073 (8)	0.0031 (7)
C3	0.0125 (9)	0.0176 (10)	0.0144 (9)	0.0013 (7)	0.0049 (8)	0.0000 (7)
C4	0.0181 (10)	0.0140 (9)	0.0175 (10)	0.0015 (7)	0.0090 (9)	0.0024 (7)
C5	0.0208 (11)	0.0230 (11)	0.0264 (12)	−0.0008 (9)	0.0084 (10)	−0.0056 (9)
C6	0.0358 (13)	0.0267 (13)	0.0335 (13)	−0.0074 (10)	0.0165 (12)	−0.0107 (10)
C7	0.0192 (11)	0.0282 (12)	0.0279 (12)	−0.0049 (9)	0.0112 (10)	−0.0022 (10)

### catena-Poly[[diaquadiimidazolecobalt(II)]-µ2-2,3,5,6-tetrabromobenzene-1,4-dicarboxylato] Geometric parameters (Å, º)

**Table:**

Br1—C3	1.890 (2)	N1—C5	1.373 (3)
Br2—C4	1.897 (2)	N2—C7	1.340 (3)
Co1—N1^i^	2.0850 (18)	N2—C6	1.363 (3)
Co1—N1	2.0850 (18)	N2—H1	0.80 (3)
Co1—O3^i^	2.1006 (16)	C1—C2	1.531 (3)
Co1—O3	2.1007 (16)	C2—C4^ii^	1.388 (3)
Co1—O1^i^	2.1678 (13)	C2—C3	1.392 (3)
Co1—O1	2.1678 (13)	C3—C4	1.392 (3)
O1—C1	1.254 (2)	C5—C6	1.364 (3)
O2—C1	1.246 (2)	C5—H5	0.9500
O3—H2	0.76 (3)	C6—H6	0.9500
O3—H3	0.75 (4)	C7—H7	0.9500
N1—C7	1.320 (3)		
			
N1^i^—Co1—N1	180.00 (11)	C7—N2—H1	124 (2)
N1^i^—Co1—O3^i^	89.06 (7)	C6—N2—H1	128 (2)
N1—Co1—O3^i^	90.94 (7)	O2—C1—O1	127.11 (18)
N1^i^—Co1—O3	90.94 (7)	O2—C1—C2	116.28 (17)
N1—Co1—O3	89.06 (7)	O1—C1—C2	116.61 (16)
O3^i^—Co1—O3	180.00 (4)	C4^ii^—C2—C3	118.53 (18)
N1^i^—Co1—O1^i^	88.31 (6)	C4^ii^—C2—C1	121.38 (18)
N1—Co1—O1^i^	91.69 (6)	C3—C2—C1	120.00 (17)
O3^i^—Co1—O1^i^	89.81 (6)	C4—C3—C2	120.56 (18)
O3—Co1—O1^i^	90.19 (6)	C4—C3—Br1	121.29 (15)
N1^i^—Co1—O1	91.69 (6)	C2—C3—Br1	118.15 (14)
N1—Co1—O1	88.31 (6)	C2^ii^—C4—C3	120.90 (18)
O3^i^—Co1—O1	90.19 (6)	C2^ii^—C4—Br2	118.19 (15)
O3—Co1—O1	89.81 (6)	C3—C4—Br2	120.90 (15)
O1^i^—Co1—O1	180.0	C6—C5—N1	109.6 (2)
C1—O1—Co1	128.92 (12)	C6—C5—H5	125.2
Co1—O3—H2	109 (2)	N1—C5—H5	125.2
Co1—O3—H3	135 (3)	N2—C6—C5	106.0 (2)
H2—O3—H3	117 (3)	N2—C6—H6	127.0
C7—N1—C5	105.28 (19)	C5—C6—H6	127.0
C7—N1—Co1	125.05 (15)	N1—C7—N2	111.6 (2)
C5—N1—Co1	129.53 (14)	N1—C7—H7	124.2
C7—N2—C6	107.5 (2)	N2—C7—H7	124.2
			
Co1—O1—C1—O2	−6.2 (3)	Br1—C3—C4—C2^ii^	−179.05 (15)
Co1—O1—C1—C2	173.49 (12)	C2—C3—C4—Br2	−178.98 (14)
O2—C1—C2—C4^ii^	−87.3 (2)	Br1—C3—C4—Br2	1.6 (2)
O1—C1—C2—C4^ii^	93.0 (2)	C7—N1—C5—C6	−0.2 (3)
O2—C1—C2—C3	89.4 (2)	Co1—N1—C5—C6	−176.08 (16)
O1—C1—C2—C3	−90.4 (2)	C7—N2—C6—C5	−0.4 (3)
C4^ii^—C2—C3—C4	−0.3 (3)	N1—C5—C6—N2	0.4 (3)
C1—C2—C3—C4	−177.08 (16)	C5—N1—C7—N2	0.0 (3)
C4^ii^—C2—C3—Br1	179.07 (14)	Co1—N1—C7—N2	176.07 (15)
C1—C2—C3—Br1	2.3 (2)	C6—N2—C7—N1	0.3 (3)
C2—C3—C4—C2^ii^	0.4 (3)		

Symmetry codes: (i) −*x*+1, −*y*+2, −*z*+2; (ii) −*x*+1/2, −*y*+5/2, −*z*+1.

### catena-Poly[[diaquadiimidazolecobalt(II)]-µ2-2,3,5,6-tetrabromobenzene-1,4-dicarboxylato] Hydrogen-bond geometry (Å, º)

**Table:**

*D*—H···*A*	*D*—H	H···*A*	*D*···*A*	*D*—H···*A*
C6—H6···Br1^iii^	0.95	3.10	3.946 (3)	149
N2—H1···O2^iv^	0.80 (3)	2.01 (3)	2.808 (2)	174 (3)
O3—H2···O2	0.76 (3)	1.99 (3)	2.700 (2)	155 (3)
O3—H3···O1^v^	0.75 (4)	2.06 (4)	2.809 (2)	176 (4)

Symmetry codes: (iii) *x*, *y*−1, *z*; (iv) −*x*+1/2, *y*−1/2, −*z*+3/2; (v) *x*, −*y*+2, *z*+1/2.
