# Supplementary material for: Crystal structure of catena-poly[[di­aqua­di­imida­zole­cobalt(II)]-μ2-2,3,5,6-tetra­bromo­benzene-1,4-di­carboxyl­ato]
Source: Acta Crystallogr E Crystallogr Commun. 2024 Oct 31;80(Pt 11):1217–20. doi: 10.1107/S2056989024009915 (PMC11660475; doi:10.1107/S2056989024009915)
Supplement: Supplementary file 4 [file e-80-01217-sup4.pdf]

# Crystal Structure of Poly[diaqua($\mu_2$-2,3,5,6-tetrabromobenzene-1,4-dicarboxylato)(diimidazole)Cobalt(II)]

Hitoshi Kumagai*[a], Satoshi Kawata[b] and Nobuhiro Ogihara[a]

[a]*Toyota Central R&D Labs., Inc., 41-1 Yokomichi Nagakute, Aichi, 480-1192, Japan*
[b]*Department of Chemistry, Fukuoka University, 8-19-1 Nanakuma, Jonan-ku, Fukuoka 814-0180, Japan*

Supporting Information

Experiment

Thermogravimetric analyses (TGA) were performed with a temperature range of 30 °C–800 °C using a Shimazu TGA-50 TG-DTA system. Infrared spectra (IR) were recorded using a Thermo Nicolet Avatar 360 FT-IR spectrophotometer. The absorption spectra of ultraviolet-visible light (UV-vis) were measured using a JASCO V-560 spectrophotometer.

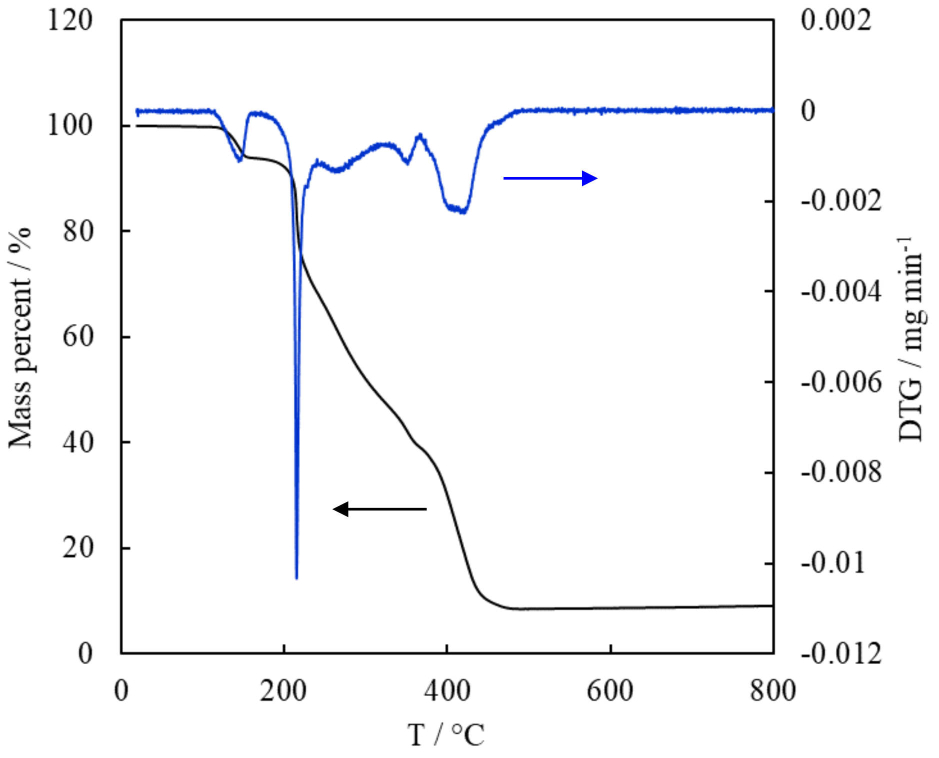


Figure S1. Thermogravimetric analysis and dTG curve for [Co($Br_4$bdc)(im)$_2$($H_2O$)$_2$].

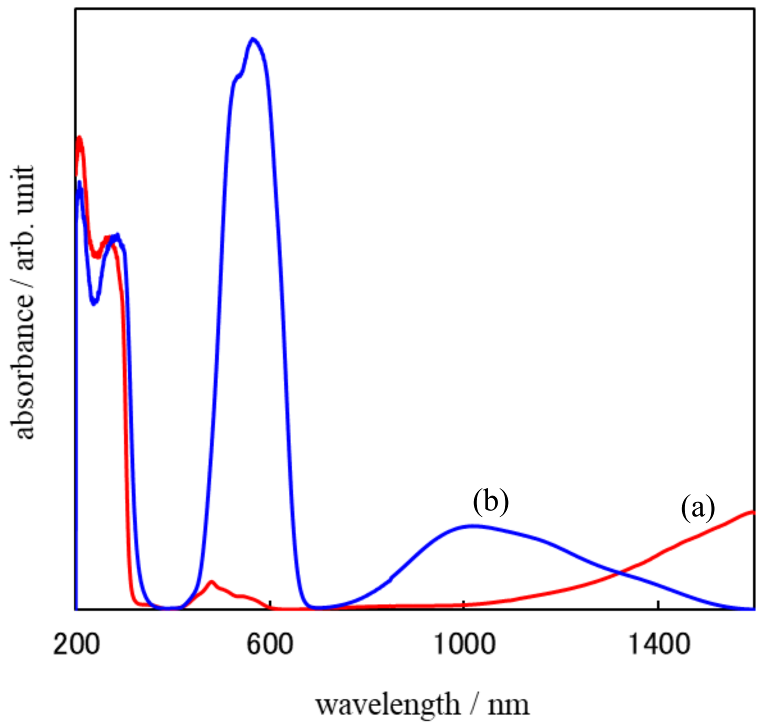


Figure S2. Electronic diffuse-reflectance spectra for (a) [Co($Br_4$bdc)(im)$_2$($H_2O$)$_2$] and (b) [Co($Br_4$bdc)(im)$_2$].

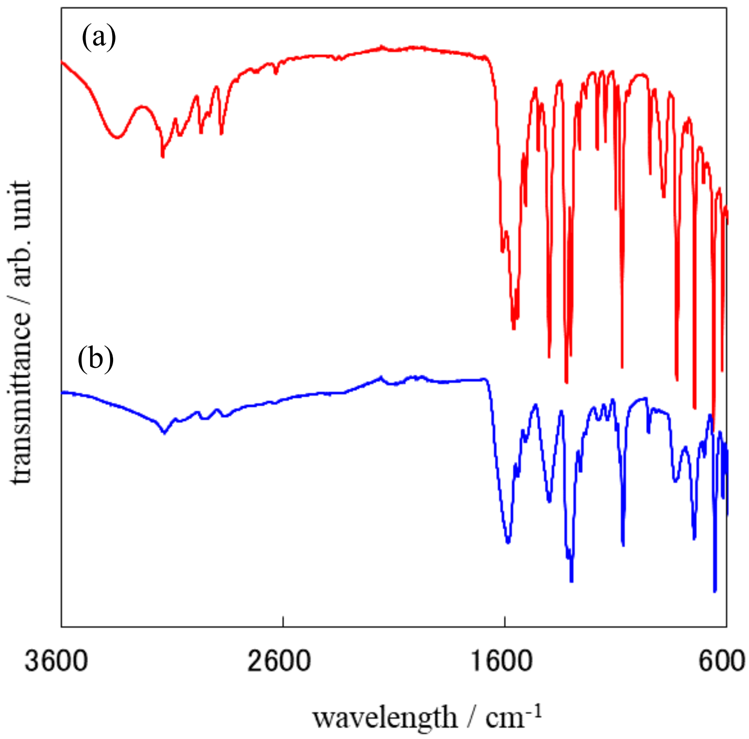


Figure S3. IR spectra for (a) [Co($Br_4$bdc)(im)$_2$($H_2O$)$_2$] and (b) [Co($Br_4$bdc)(im)$_2$].
